# Personality Profiles and Self-Efficacy Among Primary and Secondary School Teachers: A Person-Centered Analysis

**DOI:** 10.3390/bs16101883

**Published:** 2026-10-13

**Authors:** Huanxia Ding, Shu Wang

**Affiliations:** Faculty of Education, Northeast Normal University, Renmin Street 5268, Changchun 130024, China

**Keywords:** HEXACO traits, teacher self-efficacy, person-centered approach

## Abstract

This study aimed to identify HEXACO personality profiles among primary and secondary school teachers in China and examine their associations with teacher self-efficacy. Based on data from 1895 teachers, latent profile analysis was used to identify distinct personality profiles. R3STEP and BCH analyses were then conducted to examine the associations of demographic characteristics with profile membership and differences in teacher self-efficacy across profiles, respectively. Finally, structural equation modeling (SEM) was used to examine the associations between personality and teacher self-efficacy. Three distinct personality profiles were identified: Balanced, Engaged, and Resilient. Teachers in the Engaged and Resilient Profiles reported higher levels of self-efficacy than those in the Balanced Profile. Extraversion, Agreeableness, Conscientiousness, and Openness to Experience were positively associated with teacher self-efficacy. These findings provide empirical evidence for understanding personality heterogeneity among primary and secondary school teachers in China and its associations with teacher self-efficacy.

## 1. Introduction

Teacher self-efficacy refers to teachers’ beliefs in their ability to accomplish teaching tasks, manage classrooms, and promote student learning, and is an important psychological factor for understanding teachers’ instructional behavior and individual differences (Bandura, 1977; Tschannen-Moran & Woolfolk Hoy, 2001). From a social cognitive perspective, self-efficacy reflects individuals’ judgments of their capabilities to perform specific tasks and shapes their effort, persistence, and behavioral responses to task demands (Bandura, 1977). For teachers, these efficacy beliefs are closely related to teaching persistence, enthusiasm, instructional practices, and student learning outcomes (Tschannen-Moran & Woolfolk Hoy, 2001).

Research based on the Big Five has found that different personality dimensions are associated with teacher self-efficacy to varying degrees (Jamil et al., 2012; Djigić et al., 2014; Senler & Sungur-Vural, 2013). Broader evidence further indicates that teacher personality is systematically related to teaching effectiveness and other work-related outcomes (Klassen & Tze, 2014; Kim et al., 2019). Thus, personality may represent an important individual characteristic associated with teachers’ efficacy beliefs.

From a holistic perspective on personality, individual personality is not determined by isolated traits but reflects the combined configuration of multiple traits (Fleeson & Jayawickreme, 2015). However, existing research has primarily adopted a variable-centered approach, examining the relationships between individual personality dimensions and teacher self-efficacy separately (Jamil et al., 2012; Kim et al., 2019). In contrast, a person-centered approach can identify configurations of multiple personality dimensions within individuals and examine differences in relevant outcomes across these configurations (Merz & Roesch, 2011; Perera et al., 2018). This perspective also has practical implications for teacher professional development, as identifying such configurations may provide a differentiated perspective for understanding teachers’ professional strengths and potential areas for further development.

Within existing person-centered research, studies of teacher personality profiles have provided initial evidence. Previous research has found differences in teacher self-efficacy and other work-related outcomes across personality configurations (Perera et al., 2018; Franz et al., 2024; Răducu & Stănculescu, 2022). However, existing research has focused primarily on the Big Five framework, and the HEXACO model has received comparatively limited attention in personality profile research.

The HEXACO model provides a six-dimensional structure distinct from the Big Five, comprising Honesty–Humility, Emotionality, Extraversion, Agreeableness, Conscientiousness, and Openness to Experience, with Honesty–Humility constituting a distinct dimension (Ashton & Lee, 2007, 2008, 2019). Previous research has shown that HEXACO personalities can form distinct profiles characterized by different overall levels and trait configurations (Daljeet et al., 2017; She et al., 2025). Although HEXACO research has extended to diverse occupational groups, its application to primary and secondary school teachers remains relatively limited. In China, existing research has focused primarily on preservice teachers (Liu et al., 2025). The high demands placed on teachers within China’s basic education system impose considerable occupational pressure on primary and secondary school teachers, making the maintenance of teaching efficacy a sustained professional requirement. Thus, direct evidence remains limited on HEXACO-based personality profiles among in-service teachers, particularly Chinese primary and secondary school teachers, and their associations with teacher self-efficacy.

Accordingly, this study first explored whether identifiable personality profiles could be derived from the six HEXACO dimensions among Chinese primary and secondary school teachers and, if so, what characteristics these profiles would exhibit. We then examined the associations between demographic characteristics and profile membership, differences in the three dimensions of teacher self-efficacy across profiles, and the overall associations between the six HEXACO dimensions and the three dimensions of teacher self-efficacy in the full sample. Thus, the study examined the relationship between teacher personality and self-efficacy at both the personality configuration and personality dimension levels.

## 2. Methods

### 2.1. Participants and Procedures

Participants were recruited through an online questionnaire using convenience sampling. The research team contacted primary and secondary school teachers in Jilin, Henan, Guangdong, Anhui, Shanghai, Zhejiang, Liaoning, and Heilongjiang, China, who then distributed the questionnaire within their schools. The questionnaire informed participants that participation was voluntary and anonymous and that they could withdraw at any time. Of the 2278 responses collected, 383 were excluded based on prespecified data quality criteria, including excessively short completion times, consecutive selection of the same response option, and incorrect attention-check responses. As all items were required for submission, no missing data were present, yielding a final sample of 1895 teachers. The sample consisted of 465 male teachers (24.5%) and 1430 female teachers (75.5%). In terms of age, 24.6% were ≤30 years old, 34.2% were 31–40 years old, 28.9% were 41–50 years old, and 12.2% were over 50 years old. Regarding teaching experience, 21.8% had ≤5 years of experience, 20.6% had 6–10 years, 14.7% had 11–15 years, 11.7% had 16–20 years, 13.0% had 21–25 years, and 18.2% had >25 years. The participants included 595 primary school teachers (31.4%), 681 junior secondary school teachers (35.9%), and 619 senior secondary school teachers (32.7%).

### 2.2. Measures

Personality traits: The present study used the Chinese version of the HEXACO-60 to assess teachers’ personality. The original scale was developed by Ashton and Lee (2009), and the Chinese version was obtained from the official HEXACO website. The scale comprises six dimensions—Honesty–Humility, Emotionality, Extraversion, Agreeableness, Conscientiousness, and Openness to Experience—with 60 items in total and 10 items per dimension. Reverse-scored items were recoded before calculating scores for each personality dimension. Responses were rated on a 5-point Likert scale. Confirmatory factor analysis showed an acceptable model fit (χ^2^/df = 4.059, RMSEA = 0.040, SRMR = 0.046, IFI = 0.909, TLI = 0.905, CFI = 0.909).

Teacher Self-Efficacy: Teacher self-efficacy was measured using the 12-item short form of the Teachers’ Sense of Efficacy Scale (TSES) developed by Tschannen-Moran and Woolfolk Hoy (2001), comprising three dimensions: instructional strategies, classroom management, and student engagement. In the present study, the items were translated into Chinese based on the original scale and back-translated by a collaborator to ensure consistency in meaning with the original items. The original short-form TSES uses a 9-point response scale; the present study adopted a 5-point scale to align with the response formats of the other study variables and reduce respondents’ response burden. Higher scores indicate greater teacher self-efficacy. Confirmatory factor analysis showed good model fit (χ^2^/df = 4.586, RMSEA = 0.044, SRMR = 0.033, IFI = 0.985, TLI = 0.980, CFI = 0.985).

Cronbach’s alpha coefficients are presented on the diagonal of Table 1 and ranged from 0.84 to 0.91.

### 2.3. Analytical Strategies

Data were analyzed using SPSS 26.0, AMOS 26.0, and Mplus 8.3. First, Harman’s single-factor test was used to provide a preliminary assessment of common method bias (Podsakoff et al., 2003), with the first factor accounting for 21.88% of the variance. Second, latent profile analysis was conducted using standardized scores on the six HEXACO personality dimensions, and the final profile structure was determined based on model fit, classification quality, substantive interpretability, and random-split replication. Third, the R3STEP method was used to examine the associations between demographic characteristics and profile membership, and the BCH method was used to compare teacher self-efficacy across personality profiles (Asparouhov & Muthén, 2014). Finally, a structural equation model was estimated in the full sample, with all six personality dimensions entered simultaneously and gender and age controlled, to examine the overall associations between personality dimensions and the three dimensions of teacher self-efficacy.

## 3. Results

### 3.1. The Relationship Between HEXACO Traits and Teacher Self-Efficacy

As shown in Table 1, Agreeableness, Extraversion, Conscientiousness, Honesty–Humility, and Openness to Experience were positively correlated with all three dimensions of teacher self-efficacy. Emotionality was weakly negatively correlated with instructional strategy efficacy (*r* = −0.05) and student engagement efficacy (*r* = −0.07), but not with classroom management efficacy. Agreeableness was relatively strongly correlated with student engagement efficacy (*r* = 0.48) and classroom management efficacy (*r* = 0.45).

### 3.2. Latent Profiles of Teacher Personality

The fit indices for the teacher personality profile models are presented in Table 2. A latent profile analysis was conducted using standardized scores on the six HEXACO personality dimensions. As the number of profiles increased, AIC, BIC, and SSA-BIC continued to decrease, and the LMR and BLRT were significant for M2–M5, indicating improved model fit with additional profiles. Therefore, candidate models were further compared based on classification quality and substantive interpretability (Nylund-Gibson & Choi, 2018; Nylund et al., 2007). Compared with M3, M4 showed better fit indices but lower entropy (0.714 vs. 0.833). Although entropy increased to 0.772 in M5, it remained lower than that of M3, and the additional profiles did not provide sufficient substantive distinction.

The random-split analysis replicated the three-profile structure in both subsamples. The Balanced, Engaged, and Resilient Profiles accounted for 71.41%, 20.95%, and 7.65% of the first subsample (Entropy = 0.850) and 67.41%, 26.44%, and 6.15% of the second subsample (Entropy = 0.821), respectively. The six-dimensional personality patterns were also broadly consistent with those observed in the full sample, providing further support for the stability of the three-profile structure. Detailed results of the random-split validation are provided in Supplementary Tables S2 and S3.

**Figure 1:**
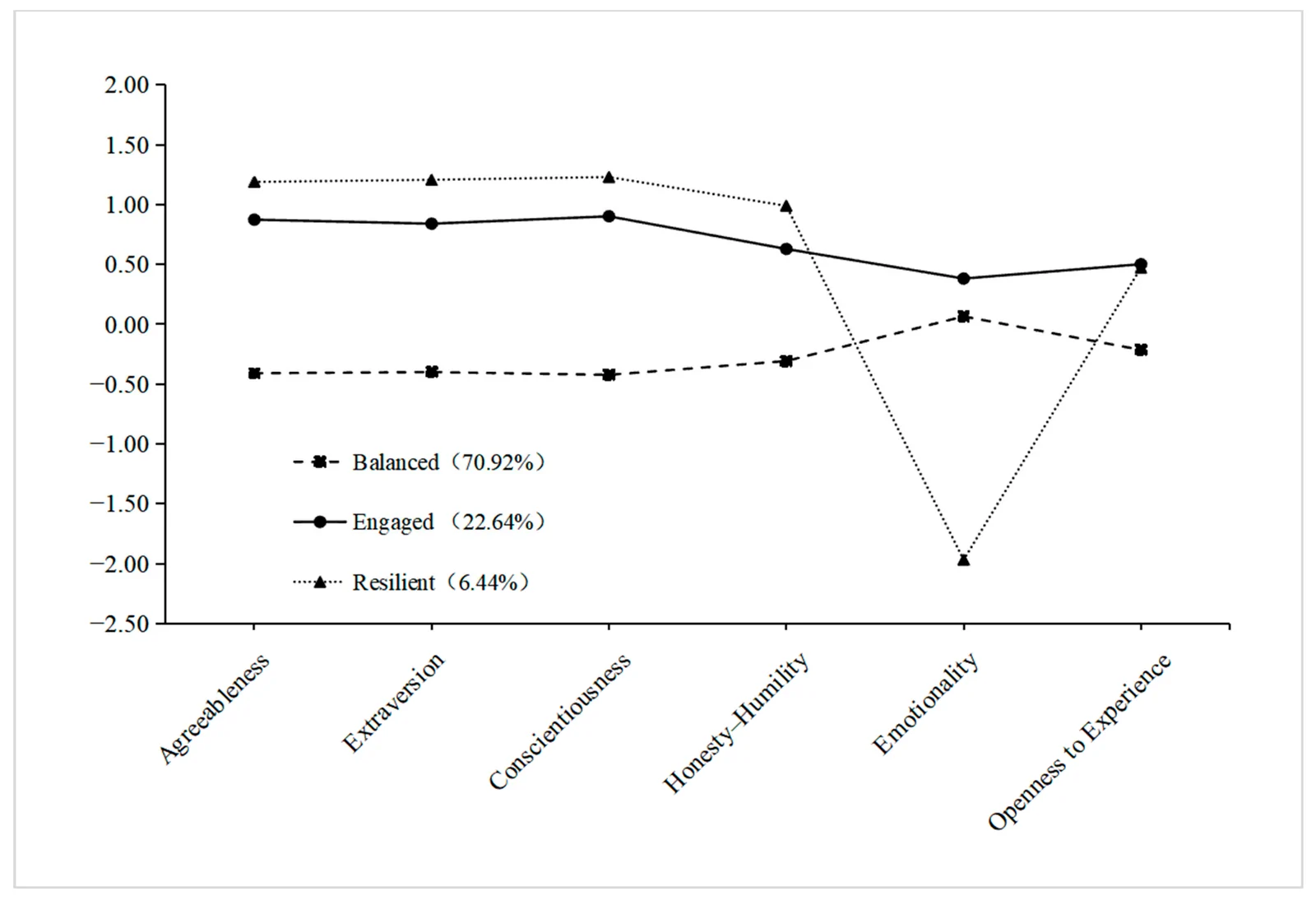
Profiles of latent classes of teacher personality based on standardized HEXACO scores.

The six-dimensional personality characteristics and profile shapes are presented in Figure 1. The first profile (*N* = 1344, 70.92%) showed scores across all personality dimensions near but slightly below the sample means, with relatively small differences across dimensions, indicating a relatively balanced personality configuration. It was therefore named the Balanced Profile. The second profile (*N* = 429, 22.64%) showed above-average scores across all dimensions, with relatively higher Conscientiousness, Agreeableness, and Extraversion and the highest Openness to Experience, and was characterized as relatively high and balanced overall. It was named the Engaged Profile. The third profile (*N* = 122, 6.44%) showed relatively high Conscientiousness, Agreeableness, Extraversion, and Honesty–Humility, above-average Openness to Experience, and substantially lower Emotionality. Its overall configuration was broadly consistent with the Resilient Profile identified in previous HEXACO studies and was therefore named the Resilient Profile (Sadiković et al., 2016). Detailed profile estimates are provided in Supplementary Table S1.

### 3.3. Demographic Characteristics and Latent Profile Membership

The associations between demographic variables and latent profile membership were examined using the R3STEP procedure, as shown in Table 3. The R3STEP analysis used the Balanced profile as the reference class and simultaneously included sex, age, teaching experience, homeroom teacher status, school level, and teaching subject as predictors. Compared with female teachers, male teachers were less likely to belong to the Engaged Profile (OR = 0.523, 95% CI [0.358, 0.764]) but more likely to belong to the Resilient Profile (OR = 1.665, 95% CI [1.052, 2.637]). Teachers with longer teaching experience were more likely to belong to the Engaged Profile (OR = 1.173, 95% CI [1.001, 1.374]). Compared with primary school teachers, Junior secondary school teachers were less likely to belong to the Engaged Profile (OR = 0.585, 95% CI [0.417, 0.820]). The remaining demographic variables were not significantly associated with latent profile membership.

### 3.4. Self-Efficacy Across Personality Profiles

To examine differences in teacher self-efficacy across latent personality profiles, the Bolck–Croon–Hagenaars method (BCH) was conducted in Mplus using robust maximum likelihood estimation (MLR). As shown in Table 4, the three latent personality profiles differed significantly across all three dimensions of teacher self-efficacy. The BCH χ^2^ values for efficacy in instructional strategies, classroom management, and student engagement were 172.12, 424.93, and 647.87, respectively (all *p* < 0.001).

Pairwise comparisons showed that the Balanced profile scored significantly lower than both the Engaged and Resilient profiles across all three dimensions. Specifically, efficacy in instructional strategies was lower in the Balanced profile (*M* = 3.80, *SE* = 0.02) than in the Engaged (*M* = 4.36, *SE* = 0.04) and Resilient (*M* = 4.35, *SE* = 0.08) profiles; efficacy in classroom management was lower in the Balanced profile (*M* = 3.90, *SE* = 0.02) than in the Engaged (*M* = 4.56, *SE* = 0.03) and Resilient (*M* = 4.50, *SE* = 0.06) profiles; and efficacy in student engagement was lower in the Balanced profile (*M* = 3.67, *SE* = 0.02) than in the Engaged (*M* = 4.45, *SE* = 0.03) and Resilient (*M* = 4.51, *SE* = 0.04) profiles. No significant differences were found between the Engaged and Resilient profiles across any of the three dimensions.

### 3.5. Associations Between Personality Traits and Teacher Self-Efficacy

Following the analysis of differences in teacher self-efficacy across personality profiles, we further examined the associations between the six personality dimensions and the three aspects of teacher self-efficacy in the full sample. Sex and age were controlled for, and robust maximum likelihood estimation (MLR) was used.

As shown in Table 5, Extraversion, Agreeableness, Conscientiousness, and Openness to Experience were positively associated with all three dimensions of teacher self-efficacy (*ps* ≤ 0.003), whereas Honesty–Humility and Emotionality were not significantly associated with any efficacy dimension (*ps* ≥ 0.095). Agreeableness showed the strongest associations, with *β* = 0.206, 0.280, and 0.295 for efficacy in instructional strategies, classroom management, and student engagement, respectively (all *ps* < 0.001). Extraversion showed the next strongest associations (*β* = 0.124, 0.173, and 0.219; all *ps* < 0.001). The associations were smaller for Conscientiousness (*β* = 0.084, 0.104, and 0.098; *ps* ≤ 0.001) and Openness to Experience (*β* = 0.067, 0.072, and 0.058; *ps* ≤ 0.003), although both were significant across all three efficacy dimensions.

The three models explained 14.8%, 25.7%, and 29.0% of the variance in efficacy in instructional strategies, classroom management, and student engagement, respectively. The explained variance was highest for student engagement.

## 4. Discussion

### 4.1. Heterogeneity in Teachers’ Personality Profiles

Primary and secondary school teachers show heterogeneous patterns formed by different combinations of personality dimensions. Among these, the Balanced Profile is characterized by a relatively balanced pattern across personality dimensions, whereas the Engaged Profile and Resilient Profile exhibit distinct patterns with different emphases. Notably, both the Engaged Profile and Resilient Profile exhibit relatively high Honesty–Humility, suggesting that moral self-regulation and modest interpersonal qualities may characterize some personality configurations among Chinese primary and secondary school teachers. A recent study of Chinese preservice teachers similarly highlighted the importance of Honesty–Humility in teacher-related characteristics (Liu et al., 2025).

R3STEP examined the associations between demographic characteristics and latent profile membership. When gender, age, teaching experience, homeroom teacher status, school level, and teaching subject were entered simultaneously, only gender, teaching experience, and school level were significant. By gender, female teachers were more likely to belong to the Engaged Profile, whereas male teachers were more likely to belong to the Resilient Profile. These differences may reflect long-term socialization and the social roles and behavioral norms associated with gender (Furnham & Treglown, 2023). Teachers with longer teaching experience were more likely to belong to the Engaged Profile, which may be related to the professional adaptation and teaching experience developed through accumulated teaching experience. Junior secondary school teachers were less likely than primary school teachers to belong to the Engaged Profile. Primary and secondary school teaching involve different instructional and interactional demands, with primary school teaching often requiring more frequent teacher–student emotional interactions (Hargreaves, 2000).

### 4.2. Differences in Teacher Self-Efficacy Across Personality Profiles

Teachers’ personality characteristics are systematically associated with work-related outcomes, including teacher self-efficacy (Kim et al., 2019). Furthermore, based on teacher personality profiles, Perera et al. (2018) found differences in self-efficacy and other work-related variables across teachers with different personality configurations, providing direct support for understanding teacher self-efficacy from an overall personality configuration perspective. The present study further found that teachers in the Engaged Profile and Resilient Profile scored significantly higher than those in the Balanced Profile on instructional strategies efficacy, classroom management efficacy, and student engagement efficacy, with no significant differences between the two profiles. The consistent pattern across all three dimensions suggests that the association between personality configurations and teacher self-efficacy may be relatively general across these efficacy dimensions.

Previous research has shown that teachers’ emotions and emotional labor are closely related to their use of instructional strategies and students’ classroom engagement (Frenzel et al., 2021; Burić & Frenzel, 2021). From an interpersonal perspective, teachers in both the Engaged and Resilient Profiles showed relatively high levels of Extraversion and Agreeableness, which may facilitate positive teacher–student interactions. From an emotional perspective, teachers in the Resilient Profile showed markedly lower Emotionality, which may reflect relatively stable emotional states and, in turn, help them maintain positive judgments of their own teaching capabilities.

### 4.3. The Relationship Between Teacher Personality and Self-Efficacy

Further regression analysis showed that, after simultaneously entering all six personality dimensions and controlling for gender and age, Extraversion, Agreeableness, Conscientiousness, and Openness to Experience were positively associated with all three dimensions of teacher self-efficacy.

Agreeableness showed the strongest associations across the three efficacy dimensions. In HEXACO, Agreeableness emphasizes tolerance, patience, gentleness, and anger control (Ashton & Lee, 2007), which are closely relevant to managing classroom conflicts, maintaining teacher–student relationships, and responding to students’ needs. High-quality teacher–student interactions also require warmth, support, and emotional stability (Pianta & Hamre, 2009). Thus, the interpersonal regulatory resources reflected in Agreeableness may be particularly relevant to teachers’ positive judgments of their teaching capabilities.

Notably, Kim et al. (2018) identified Agreeableness as the strongest personality predictor of teacher personal support, suggesting its relevance to teachers’ interpersonal and supportive functions. In contrast, Kim et al. (2019) found no significant association between Agreeableness and teacher effectiveness within the Big Five framework, whereas the present study identified Agreeableness as one of the strongest personality correlates of teacher self-efficacy within the HEXACO framework. This difference may reflect how the two models conceptualize Agreeableness: HEXACO incorporates anger control and interpersonal tolerance into Agreeableness while separating moral traits such as honesty and humility into Honesty–Humility, potentially capturing personality resources more directly relevant to teachers’ classroom interactions and management.

Extraversion showed the next strongest associations. Its social confidence, social vitality, and active interaction tendency (Ashton & Lee, 2007) may support positive instructional interactions relevant to teacher efficacy. Conscientiousness and Openness to Experience showed relatively weaker associations. The former reflects planning and goal persistence, whereas the latter reflects cognitive flexibility and openness to new experiences; both are more relevant to task-oriented aspects of teaching, such as instructional organization and innovation (Perera et al., 2018).

Finally, the model explained the largest proportion of variance in student engagement efficacy, possibly because it depends more strongly on teacher–student relationships and classroom interactions, which are closely related to Agreeableness and Extraversion.

### 4.4. Practical Implications and Limitations

First, teacher personality provides useful information for understanding individual differences in teachers’ self-efficacy. This may help schools provide more targeted professional support for teachers. Personality-related characteristics and professional attributes can be assessed through evidence-based approaches, such as situational judgment tests (Klassen & Kim, 2021; Nadmilail et al., 2023), providing information that may help schools better understand teachers’ individual characteristics and support differentiated professional development. Second, teacher professional development should promote the complementarity of different personality resources through peer collaboration, team-based professional learning, and project-based teaching. Finally, as AI becomes increasingly integrated into lesson preparation, student learning analysis, and feedback, schools need to reconsider the core value and irreplaceable aspects of teachers’ work (UNESCO, 2025). More attention could be given to aspects of teaching that rely on sustained interpersonal engagement, such as student engagement and individualized support, which remain important areas of teachers’ professional roles. Given the relatively strong associations of Agreeableness and Extraversion with student engagement efficacy, teacher development should focus not only on technological competence but also on strengthening teachers’ professional roles in interpersonal interaction and student development.

This study has several limitations. First, the convenience sample from eight regions of China is not nationally representative; the generalizability of the findings may be limited to teacher populations with characteristics similar to those of the present sample. Second, the Resilient Profile represented a relatively small proportion of the sample. Although the random-split analysis supported the three-profile structure, its stability requires further validation in larger samples from diverse regions. Third, both personality and teacher self-efficacy were assessed through teacher self-reports, which may introduce common method bias. The cross-sectional design also precludes causal inferences, and future research could incorporate multiple data sources and longitudinal designs for further validation. Finally, this study focused primarily on the relationship between personality, its configurations, and teacher self-efficacy, without considering other potentially relevant contextual factors. Future research could incorporate additional potential predictors.

## 5. Conclusions

This study identified three personality profiles among primary and secondary school teachers—Balanced, Engaged, and Resilient—highlighting heterogeneity in personality configurations. Gender, teaching experience, and school level were associated with profile membership. Teachers in the Engaged and Resilient profiles reported higher self-efficacy in instructional strategies, classroom management, and student engagement than those in the Balanced profile, with no significant differences between the former two profiles. At the trait level, Extraversion, Agreeableness, Conscientiousness, and Openness to Experience were positively associated with all three dimensions of teacher self-efficacy. Overall, this study contributes to understanding the associations between teacher personality and self-efficacy at both the personality configuration and trait levels.

## Figures and Tables

**Table 1:** Means, standard deviations, and correlations among study variables.

Variable	M	SD	Sex	Age	Teaching Experience	1	2	3	4	5	6	7	8	9
Sex	0.25	0.43	—											
Age	2.29	0.97	0.15 **	—										
Teaching experience	3.36	1.95	0.15 **	0.90 **	—									
1. Agreeableness	3.87	0.59	0.01	0.14 **	0.15 **	(0.90)								
2. Extraversion	3.75	0.55	0.03	0.11 **	0.11 **	0.54 **	(0.89)							
3. Conscientiousness	3.86	0.54	−0.04	0.14 **	0.14 **	0.43 **	0.45 **	(0.91)						
4. Honesty–Humility	3.88	0.61	−0.06 **	0.12 **	0.13 **	0.38 **	0.30 **	0.43 **	(0.91)					
5. Emotionality	3.32	0.67	−0.21 **	−0.11 **	−0.10 **	−0.15 **	−0.13 **	−0.09 **	−0.08 **	(0.90)				
6. Openness to Experience	3.46	0.66	−0.03	0.00	0.00	0.24 **	0.28 **	0.24 **	0.12 **	0.00	(0.89)			
7. Efficacy in Instructional Strategies	3.97	0.74	0.00	0.11 **	0.10 **	0.34 **	0.30 **	0.26 **	0.18 **	−0.05 *	0.17 **	(0.88)		
8. Efficacy in Classroom Management	4.10	0.64	−0.02	0.13 **	0.13 **	0.45 **	0.40 **	0.34 **	0.24 **	−0.04	0.22 **	0.51 **	(0.84)	
9. Efficacy in Student Engagement	3.91	0.77	−0.01	0.12 **	0.13 **	0.48 **	0.44 **	0.35 **	0.24 **	−0.07 **	0.22 **	0.47 **	0.53 **	(0.87)

Note. Cronbach’s alpha coefficients are presented in parentheses along the diagonal. Sex was coded as 1 = male, 0 = female. * *p* < 0.05, ** *p* < 0.01.

**Table 2:** Fit indices for latent profile analysis of HEXACO personality dimensions.

Model	Class	LL	FP	AIC	BIC	SSA-BIC	LMR (*p*)	BLRT (*p*)	Entropy	Class Probabilities
M1	1	−10,342.168	12	20,708.336	20,774.900	20,736.776	—	—	—	
M2	2	−9470.437	19	18,978.874	19,084.266	19,023.903	<0.001	<0.001	0.814	72.82%/27.18%
M3	3	−9339.399	26	18,730.798	18,875.020	18,792.418	<0.001	<0.001	0.833	70.92%/22.64%/6.44%
M4	4	−9229.581	33	18,525.162	18,708.212	18,603.371	<0.001	<0.001	0.714	21.69%/6.60%/55.88%/15.83%
M5	5	−9153.801	40	18,387.601	18,609.480	18,482.401	0.003	<0.001	0.772	6.55%/34.30%/9.87%/31.08%/18.21%

**Table 3:** Demographic predictors of latent profile membership.

Predictor	Engaged vs. Balanced OR (95% CI)	*p*	Resilient vs. Balanced OR (95% CI)	*p*
Male (vs. female)	0.523 [0.358, 0.764]	0.001	1.665 [1.052, 2.637]	0.030
Age	1.009 [0.733, 1.389]	0.954	1.132 [0.673, 1.902]	0.640
Teaching experience	1.173 [1.001, 1.374]	0.049	1.197 [0.918, 1.561]	0.185
Homeroom teacher (vs. non-homeroom teacher)	0.933 [0.694, 1.256]	0.649	1.028 [0.646, 1.638]	0.906
Junior secondary school (vs. primary school)	0.585 [0.417, 0.820]	0.002	0.688 [0.392, 1.208]	0.193
Senior secondary school (vs. primary school)	0.793 [0.552, 1.139]	0.210	1.175 [0.661, 2.087]	0.583
Exploratory subject (vs. foundational Subjects)	0.953 [0.634, 1.432]	0.816	0.687 [0.360, 1.311]	0.255
Enrichment Subjects (vs. foundational Subjects)	1.362 [0.952, 1.947]	0.091	1.523 [0.879, 2.638]	0.133

Note. Subjects were classified into three categories based on their function: Foundational Subjects (Chinese, Mathematics, Foreign Languages), Exploratory Subjects (Physics, Chemistry, Biology, Elementary Science, History, Geography), and Enrichment Subjects (Politics/Morality and Law, Physical Education, Music, Art, Psychology, Labor, Information Technology).

**Table 4:** BCH Comparisons of teacher self-efficacy across latent profiles.

Teacher Self-Efficacy	Balanced	Engaged	Resilient	Overall BCH χ^2^ (df = 2)	Pairwise Comparisons
Efficacy in instructional strategies	3.80 (0.02)	4.36 (0.04)	4.35 (0.08)	172.12 ***	1 < 2; 1 < 3
Efficacy in classroom management	3.90 (0.02)	4.56 (0.03)	4.50 (0.06)	424.93 ***	1 < 2; 1 < 3
Efficacy in student engagement	3.67 (0.02)	4.45 (0.03)	4.51 (0.04)	647.87 ***	1 < 2; 1 < 3

Note. Values are BCH-adjusted estimated means (SE). 1 = Balanced; 2 = Engaged; 3 = Resilient. *** *p* < 0.001.

**Table 5:** Associations between HEXACO dimensions and teacher self-efficacy.

Predictor	Efficacy in Instructional Strategies				Efficacy in Classroom Management				Efficacy in Student Engagement			
	*β*	*SE*	*p*	95% CI	*β*	*SE*	*p*	95% CI	*β*	*SE*	*p*	95% CI
AG	0.206	0.033	<0.001	[0.142, 0.270]	0.280	0.028	<0.001	[0.226, 0.334]	0.295	0.028	<0.001	[0.240, 0.351]
EX	0.124	0.027	<0.001	[0.071, 0.178]	0.173	0.029	<0.001	[0.116, 0.230]	0.219	0.026	<0.001	[0.167, 0.270]
CO	0.084	0.026	0.001	[0.033, 0.135]	0.104	0.023	<0.001	[0.059, 0.148]	0.098	0.025	<0.001	[0.050, 0.146]
HH	0.014	0.025	0.582	[−0.036, 0.063]	0.021	0.024	0.394	[−0.027, 0.068]	0.005	0.022	0.820	[−0.038, 0.049]
EM	0.011	0.026	0.674	[−0.039, 0.061]	0.039	0.023	0.095	[−0.007, 0.084]	0.015	0.020	0.456	[−0.024, 0.054]
OP	0.067	0.022	0.002	[0.024, 0.110]	0.072	0.022	0.001	[0.029, 0.116]	0.058	0.019	0.003	[0.020, 0.096]
Sex	−0.006	0.023	0.792	[−0.051, 0.039]	−0.023	0.022	0.291	[−0.065, 0.019]	−0.019	0.021	0.364	[−0.061, 0.022]
Age	0.055	0.022	0.013	[0.011, 0.099]	0.067	0.021	0.001	[0.026, 0.108]	0.047	0.021	0.025	[0.006, 0.088]
R^2^	0.148				0.257				0.290			

Note. All coefficients are STDYX standardized. AG = Agreeableness; EX = Extraversion; CO = Conscientiousness; HH = Honesty–Humility; EM = Emotionality; OP = Openness to Experience. Sex was coded as 1 = male, 0 = female.

## Data Availability

The data that support the findings of this study are available upon request from the corresponding author.

## Supplementary Materials

The following supporting information can be downloaded at: https://www.mdpi.com/article/10.3390/bs16101883/s1, Table S1: Raw and Standardized Means of HEXACO Dimensions Across Latent Profiles; Table S2: Random-Split Validation of the Three-Profile LPA Solution; Table S3: HEXACO Dimension Means Across the Full Sample and Random Subsamples.
