# Supplementary material for: Personality Profiles and Self-Efficacy Among Primary and Secondary School Teachers: A Person-Centered Analysis
Source: Behav Sci (Basel). 2026 Oct 13;16(10):1883. doi: 10.3390/bs16101883 (PMC13701774; doi:10.3390/bs16101883)
Supplement: Supplementary file 1 [file behavsci-16-01883-s001.zip › behavsci-4530992-supplementary.pdf]

Supplementary Materials

Table S1. Raw and Standardized Means of HEXACO Dimensions Across Latent Profiles.

HEXACO dimension	Balanced	Engaged	Resilient	Pairwise comparisons
Agreeableness	3.628 (0.017) / −0.410	4.386 (0.036) / 0.873	4.573 (0.043) / 1.188	1 < 2***; 1 < 3***; 2 < 3***
Extraversion	3.526 (0.015) / −0.400	4.205 (0.031) / 0.839	4.406 (0.048) / 1.206	1 < 2***; 1 < 3***; 2 < 3***
Conscientiousness	3.628 (0.015) / −0.423	4.339 (0.038) / 0.901	4.515 (0.047) / 1.229	1 < 2***; 1 < 3***; 2 < 3***
Honesty-Humility	3.688 (0.018) / −0.308	4.261 (0.031) / 0.628	4.482 (0.056) / 0.988	1 < 2***; 1 < 3***; 2 < 3***
Emotionality	3.365 (0.016) / 0.065	3.575 (0.045) / 0.381	2.009 (0.073) / −1.968	1 < 2***; 1 > 3***; 2 > 3***
Openness to Experience	3.314 (0.017) / −0.215	3.788 (0.047) / 0.501	3.769 (0.103) / 0.472	1 < 2***; 1 < 3***; 2 = 3 ns

Note. Values are presented as raw mean (SE) / standardized mean. Pairwise comparisons were based on raw-score profile means estimated from the latent profile model. 1 = Balanced; 2 = Engaged; 3 = Resilient. *** $p < 0.001$; ** $p < 0.01$; ns = not significant.

Table S2. Random-Split Validation of the Three-Profile LPA Solution.

Sample	N	Balanced	Engaged	Resilient	Entropy
Full sample	1,895	70.92%	22.64%	6.44%	0.833
Random subsample 1	961	71.41%	20.95%	7.65%	0.850
Random subsample 2	934	67.41%	26.44%	6.15%	0.821

Table S3. HEXACO Dimension Means Across the Full Sample and Random Subsamples.

Profile	Sample	AG	EX	CO	HH	EM	OP
Balanced	Full sample	-0.410	-0.4	-0.423	-0.308	0.065	-0.215
	N = 961	-0.367	-0.445	-0.421	-0.287	0.054	-0.218
	N = 934	-0.472	-0.364	-0.418	-0.331	0.083	-0.213
Engaged	Full sample	0.873	0.839	0.901	0.628	0.381	0.501
	N = 961	0.882	0.844	1.017	0.682	0.462	0.616
	N = 934	0.880	0.834	0.769	0.562	0.303	0.391
Resilient	Full sample	1.188	1.206	1.229	0.988	-1.968	0.472
	N = 961	1.137	1.212	1.094	0.840	-1.842	0.512
	N = 934	1.232	1.218	1.339	1.172	-2.115	0.440
